# Screening of Filamentous Fungi to Identify Biocatalysts for Lupeol Biotransformation

**DOI:** 10.3390/molecules15096140

**Published:** 2010-09-01

**Authors:** Tatiane C. de Carvalho, Aline M. Polizeli, Izabel C. C. Turatti, Marcela E. Severiano, Carlos E. de Carvalho, Sérgio R. Ambrósio, Antônio E. M. Crotti, Uir S. de Figueiredo, Paulo C. Vieira, Niege A. J. C. Furtado

**Affiliations:** 1 Faculdade de Ciências Farmacêuticas de Ribeirão Preto, Universidade de São Paulo, Ribeirão Preto, SP, 14040-903, Brazil; E-Mails: carvalho_tc@yahoo.com.br (T.C.C.); alinepolizeli@gmail.com (A.M.P.); izcristu@usp.br (I.C.C.T.); 2 Núcleo de Pesquisa em Ciências Exatas e Tecnológicas, Universidade de Franca, Franca, SP, 14404-600, Brazil; E-Mails: marcellaes@hotmail.com (M.E.S.); denossauro7@gmail.com (C.E.C.); sergioambrosio@unifran.br (S.R.A.); millercrotti@unifran.br (A.E.M.C.); 3 Instituto de Ciências Exatas e da Terra, Universidade Federal de Mato Grosso, MT, 78060-900, Brazil; E-Mail: uirs@terra.com.br (U.S.F.); 4 Centro de Ciências Exatas e de Tecnologia, Universidade Federal de São Carlos, SP, 13565-905, Brazil; E-Mail: paulo@dq.ufscar.br (P.C.V.)

**Keywords:** biocatalysts, biotransformation, filamentous fungi, lupeol, mass spectrometry

## Abstract

The goal of the study was to evaluate the ability of filamentous fungi to biotransform the pentacyclic triterpene lupeol. The microbial transformations were carried out in shake flasks in different media. Experiments were also run with control flasks. Samples of each culture were taken every 24 hours, extracted with ethyl acetate, and analyzed by GC-MS. The biotransformation of lupeol by *Aspergillus ochraceus* and *Mucor rouxii* afforded two compounds in each culture, which were detected in the cultures developed for more than seven days only in the Koch’s K1 medium. The obtained data demonstrated that *A. ochraceus* is a good biocatalyst to introduce double bonds in the lupeol structure, whereas *M. rouxii* exhibits ability to biocatalyze oxygen insertions in that pentacyclic triterpene. Mass spectrometry was demonstrated to be an efficient analytical method to select promising biocatalysts for the compound investigated in this study. The biotransformation processes were influenced by the culture medium and incubation period. The obtained results open the perspective of using *A*. *ochraceus* and *M. rouxii* in pentacyclic triterpene biotransformations.

## 1. Introduction

Triterpenes are among the most abundant natural products in the plant kingdom and exhibit huge structural diversity [1]. Among them, pentacyclic triterpenes of the lupane type, such as lupeol, betulin and betulinic acid, have been reported to have interesting bioactivities including antiviral, in particular against to human immunodeficiency virus [2,3], herpes simplex virus [4], and Epstein-Barr virus [5], anti-inflammatory [6] and antitumor against human melanoma and other types of human malignancies [7,8].

Even though more than 100 triterpene skeletons have been reported in Nature [9], hundreds of new derivatives have been synthesized [3,10]. Modifications of the parent structure may produce new potentially interesting derivatives with better pharmacokinetic properties [3], new mechanisms of action [11] and fewer unwanted side effects [12]. Bevirimat, which has entered clinical phase II evaluation in patients, for example, is a derivative of betulinic acid with anti HIV-1 activity [13,14]. NVX-207, another betulinic acid derivative with antitumor activity, is considered a candidate well suited for clinical development [15]. Most of these derivatives are obtained by synthetic approaches [10], although biotransformation processes have also been used in order to target positions more difficult to functionalize by chemical methods. 

**Figure 1:**
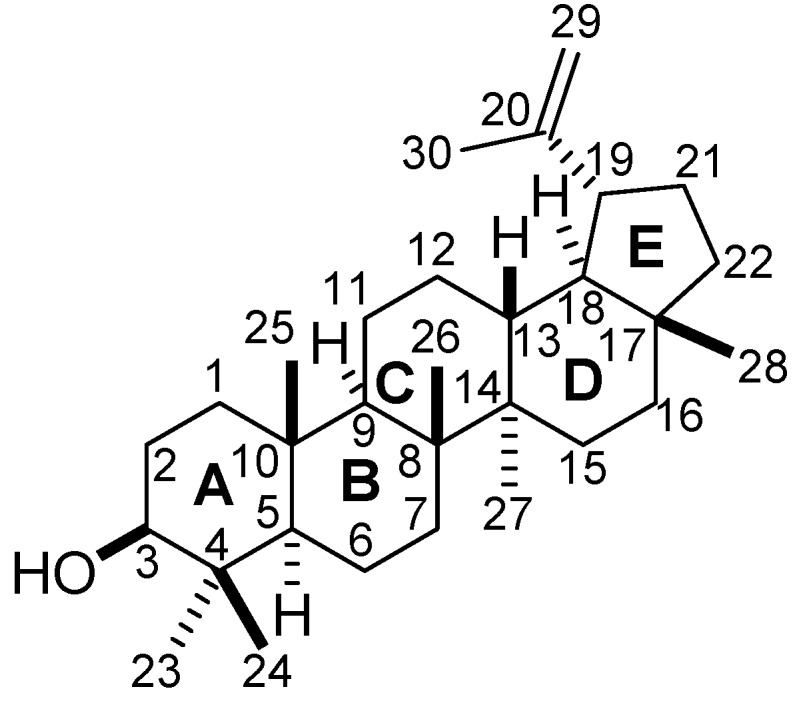
Chemical structure of lupeol.

A number of betulin, betulinic and betulonic acids derivatives have been produced by biotransformation processes [16,17], but no previous studies on the biotransformation of the triterpene lupeol (Figure 1) have been reported. Lupeol is produced by many plants and, hence, is more available than betulin, betulinic and betulonic acids [18]. 

Compared to chemical reagents microbial processes have the advantage of specificity, and of operation at relatively low temperatures without the requirement for potentially polluting heavy-metal catalysts [19]. Pure enantiomers have been produced by biotransformation processes, which are considered as a cheap and environmentally friendly alternative to chemical synthesis reactions [20]. 

Filamentous fungi are versatile and robust organisms having enormous potential for biotransformation of a variety of substrates [21,22]. Thus, the goal of this study was to evaluate the ability of a collection of filamentous fungi to biotransform the pentacyclic triterpene lupeol using different medium constituents, since literature data highlighted the need to evaluate different culture conditions in order to identify efficient biocatalysts.

## 2. Results and Discussion

The following filamentous fungi were found capable of biotransforming lupeol: *Aspergillus ochraceus* and *Mucor rouxii*. The abilities of these fungi were demonstrated only when the fungi cultures were developed in the Koch’s K1 medium. In addition, the biotransformation products were only detected after a specific incubation time, indicating the influence of the incubation time on the biotransformation reactions. 

Medium composition usually plays an important role in biotransformation processes, since different nutrients may affect the functional and structural development of the fungi. In addition, the type and concentration of cosolvents used to dissolve a water insoluble substrate in the culture medium may affect the microorganism growth, as well as the bioconversion yield. Substrate addition should be always performed in such a way that exactly the same amount of substrate, dissolved in the cosolvent, should be added to the cultures, at a concentration previously determined, in order to avoid toxicity problems. 

The time course of the biotransformation process is another key issue and is dependent on the growth characteristics of the microorganisms, the culture conditions, the substrate concentration and the type of reaction. 

The seed medium was used in order to increase the biomass of the cultures and only after the end of the growth phase, the biomass of each culture was transferred to Czapek and Koch’s K1 media, which were used in the biotransformation step. The choice of this strategy is due to the fact that the enzymes produced during the stationary phase are less specific than those produced during the growth phase. The glycosyltransferases produced in the secondary metabolism, for example, are capable of catalysing reactions with a larger number of substrates when compared with those produced in the primary metabolism [23]. The obtained results suggest that the protocol presented here is useful, but should be performed in different biotransformation media. 

The biotransformation of lupeol by *A. ochraceus* and *M. rouxii* afforded two compounds in each culture, which were detected in the cultures developed for more than seven days. These compounds were the only derivatives detected under GC-MS operating conditions. The GC retention times (RT) of the produced derivatives varied according to the fungus, as well as the relative intensities of some ions, including the molecular ion. The GC retention time of lupeol was 35.4 min. 

**Figure 2:**
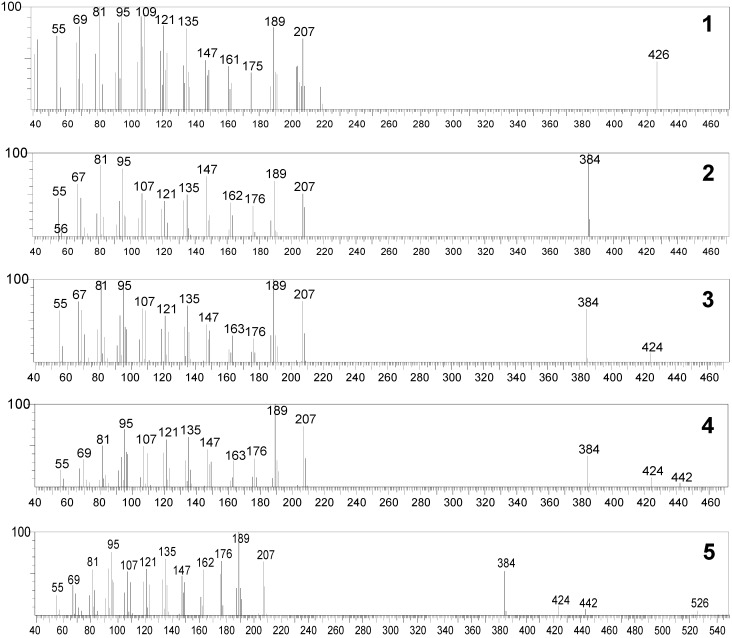
EI-MS spectra (35 eV) of lupeol (**1**) and its derivatives produced by *A. ochraceus* (**2** and **3**, retention times of 46.5 min and 48.8 min, respectively), and by *M. rouxxi* (**4** and **5**, retention times of 46.0 and 48.0 min).

The mass spectra of lupeol and its biotransformation products were initially obtained at 70 eV, which is the electron beam energy used for most spectrum libraries. However, at this energy, the mass spectra of all the biotransformation products were identical, in despite of their different retention times. Peaks of the molecular ions of such compounds could be observed only when the beam energy was set at 35 eV. The mass spectra obtained displayed a series of ions similar to those observed for lupeol, such as the fragment ions with *m/z* 189 and *m/z* 207, which is considered characteristic for the fragmentation of triterpenes with a lupane skeleton bearing a hydroxyl group in position 3 [24]. The electron ionization mass spectra of lupeol (RT = 35.468) and of the derivatives **2** and **3** produced by *A. ochraceus* (retention times of 46.5 and 48.8 min) and those produced by *M. rouxii* (**4** and **5**, retention times of 46.0 and 48.0 min) are shown in Figure 2. 

**Figure 3:**
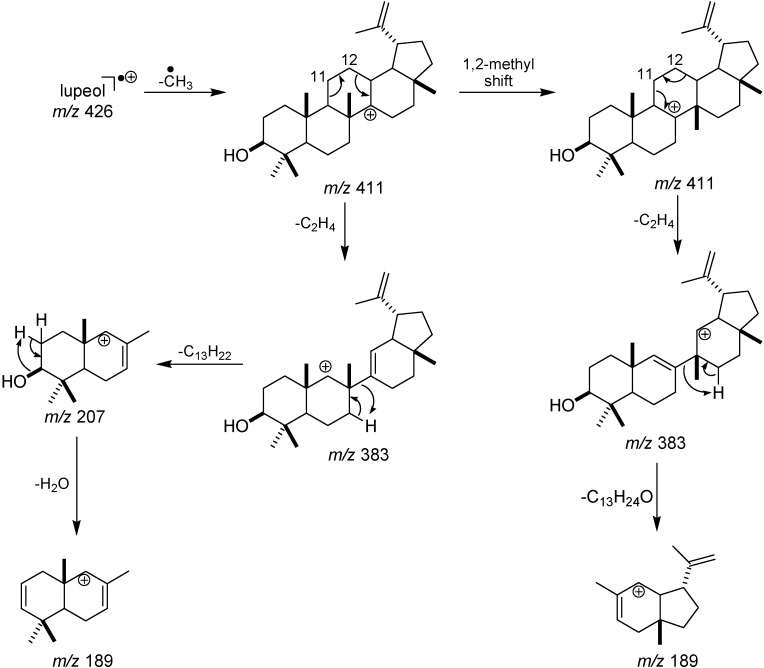
Formation of the fragment ions with *m/z* 207 and *m/z* 189, proposed with basis on [24,30].

The identification of the biotransformation products mostly have been made on the basis of nuclear magnetic resonance (NMR) data [25,26]. However, due to the complexity of the reaction media obtained from the fungi used in this study and the small amounts of the obtained extracts (range from 5 to 10 mg), the isolation of the lupeol derivatives and their characterization by NMR was not possible. Hyphenated techniques, such as gas chromatography mass spectrometry (GC-MS) and liquid chromatography mass spectrometry (LC-MS) have played a key role in the analysis of low amount of complex samples that require separation previously to the identification [27,28]. GC-MS takes the advantage of the possibility of fast identification based on the comparison with library spectra obtained at 70 eV [29], reason for why this technique was used herein. Furthermore, the fragmentation pathways of lupeol and other pentacyclic triterpenes using electron ionization mass spectrometry (EI-MS) have been extensively reported in the literature [24,30,31,32,33]. According to those studies, the fragmentation of lupeol is initiated by C-14–C-27 cleavage and consequent ^•^CH_3_ elimination, as shown in Figure 3. The fragment ions *m/z* 411 and *m/z* 383 can eventually not be observed, once they can easily decompose in other fragment ions with lower *m/z*. Otherwise, the fragment ions *m/z* 207 and *m/z* 189 have been proposed to be formed as a result of two competitive pathways, which can be used to diagnose the presence of substituents in A, B, C, D or E rings.

MS spectra of the biotransformation products of lupeol produced by *A. ochraceus* and *M. rouxii* were initially obtained at 70 eV, and hence compared with the MS spectrum of lupeol, as previously discussed in this paper. At this energy, all the MS spectra were very similar in the *m/z* range from 55 to 207. However, the MS spectra of the biotransformation products differ from the corresponding spectrum of lupeol due to the presence of an ion with *m/z* 384 in place of *m/z* 426. In principle, this could indicate that these biotransformation products are isomers with molecular weight 384, but exhibiting very different retention times in GC. Otherwise, this could also indicate that the molecular ions of such compounds decomposes into fragment ions with lower *m/z* during the flight from the ion source to the analyzer due to the high residual energy content that is transferred to the molecular ion [34]. Thus, in order to confirm the molecular weight of these compounds, MS spectrum of lupeol and its biotransformation products were obtained using lower electron beam energy. The maximum energy that allowed observing the molecular ions of such compounds was found to be 35 eV. At this energy, the fragmentation pattern of lupeol was observed to be very similar to that of 70 eV, although minor fragment ions, such as M^•+^–^•^CH_3_ and M^•+^–^•^CH_3_-C_2_H_4_ have not been observed. These spectra revealed that the biotransformation products of lupeol exhibit different molecular ions and structures, as discussed below. 

**Figure 4:**
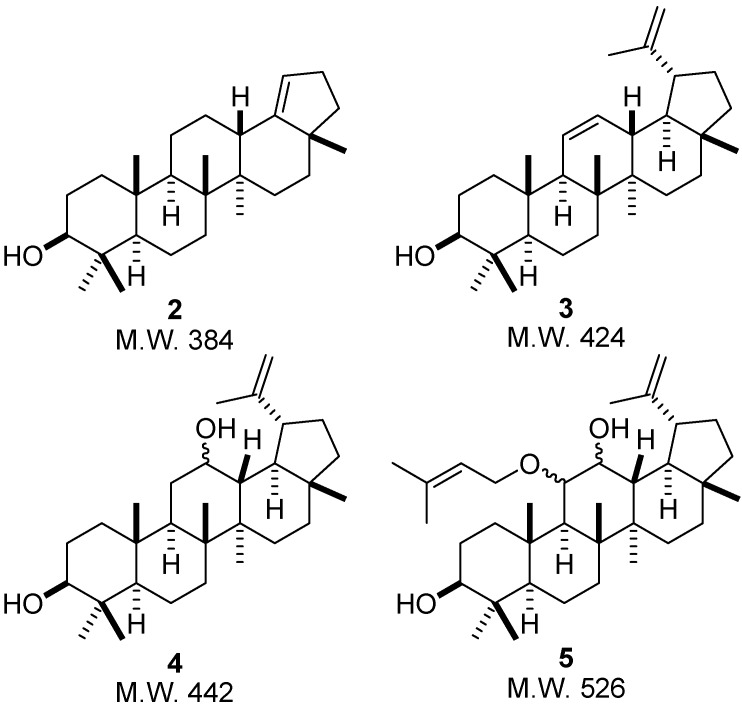
Proposed structures for the biotransformation products of lupeol, produced by *A. ochraceus* (**2** and **3**) and *M. rouxii* (**4** and **5**).

Compounds **2** and **3** were produced by *A. ochraceus* and exhibited retention times of 46.5 and 48.8 min, respectively. Analysis of the MS spectrum of compound **2** (Figure 2) revealed that its molecular ion (*m/z* 384) is 42 mass units lower than lupeol. This mass difference can be due to the elimination of the isopropenyl group at C-19 as propene and subsequent formation of a double bond between C-18 and C-19 (Figure 4). This structural modification can be supported by the intensity of the fragment ion *m/z* 147 in the MS spectrum of compound **2**, which is higher than in the MS spectrum of compounds **3**, **4**, **5** and lupeol (see Figure 2). Eliminations of alkyl groups and other size chain have been previously reported to be promoted by this fungus [35]. Otherwise, the molecular ion of compound **3** (*m/z* 424) is two mass units lower than that of lupeol (*m/z* 426), which can be associated with the formation of a double bond. Taking into account that the fragment ions with *m/z* 207 and *m/z* 189 did not show mass decrease in comparison with those of lupeol, it is proposed that the double bond is formed between C-11 and C-12 of ring C, as shown in Figure 3**. **The presence of a double bond in A, B, D or E rings would result in the formation of fragment ions with *m/z* 205 and *m/z* 187, which are not observed in the spectrum of compound **3**. Desaturation by double bond formation has been reported to be promoted by other fungi [36], but this has not been reported for *A. ochraceus*. 

Compounds **4** and **5** were produced by *M. rouxii* and exhibited retention times of 46.0 and 48.0 min, respectively. The MS spectrum of compound **4** is very similar to that of lupeol, however, its molecular ion (*m/z* 442) is 16 Da higher than lupeol. This indicates that compound **4** contains an oxygen atom more than lupeol. The presence of the fragment ions *m/z* 207 and *m/z* 189 lead us to suggest that this atom is likely bound at C-11 or C-12 of the ring C moiety, as shown in Figure 3. Finally, the molecular ion of compound **5** (*m/z* 526) is 100 mass units higher than the corresponding ion of lupeol (*m/z* 426). This mass difference is proposed to be due to two addition oxygen atoms and to a prenyl group. Hydroxylation by *Mucor* *rouxii* has been previously reported [37] and genes involved in isoprenoid biosynthesis were isolated from *Mucor circinelloides* [38]. The fragmentation pattern of compound **5** is similar to that of lupeol, despite differences between their structures. This similarity, together with the ions *m/z* 207 and *m/z* 189, can indicate that both the oxygen and the isopentenyl groups are also in the C ring, as shown in Figure 4. 

**Figure 5:**
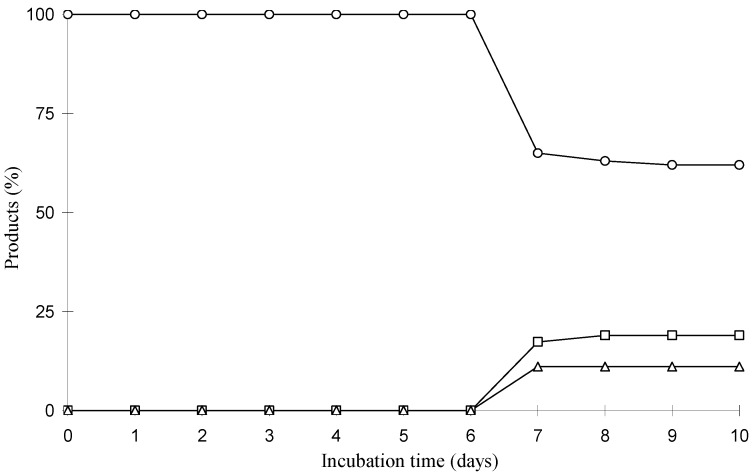
Time course in the biotransformation of lupeol by *A. ochraceus*: **(○)** lupeol; **(□)** compound **2**; (**△** ) compound **3**.

**Figure 6:**
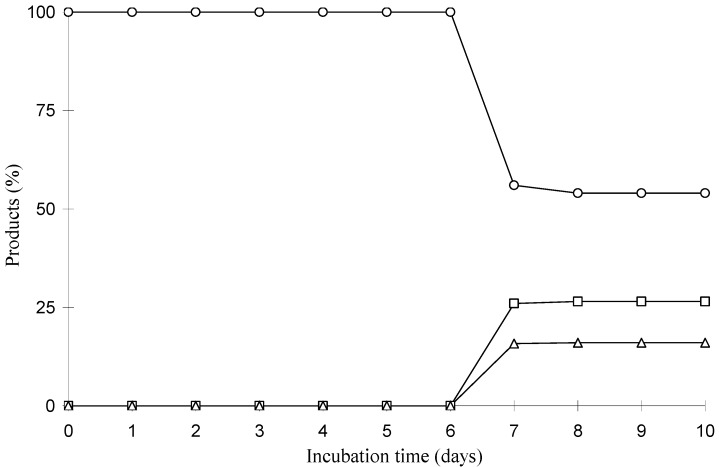
Time course in the biotransformation of lupeol by *M. rouxii*: **(○)** lupeol; **(□)** compound **4**; (**△**) compound **5**.

The time courses of these biotransformations were followed by GC-MS and the relative ratio between the substrate and its transformed products was determined on the basis of the peak area of GC. Biotransformation experiments were carried out for 240 h and each flask was taken every 24 h, extracted with ethyl acetate, and analyzed by GC-MS. The results are reported in Figure 5 and Figure 6. Lupeol was 38.0% and 46.0% consumed by *A. ochraceus* and *M. rouxii*, respectively, in 10 days. The relative ratios of compounds **2** and **3** with respect to lupeol were 17.3% and 11.1%, respectively, after 7 days incubation, increasing to 19.0% of compound **2** and remaining unchanged until the 10th day. 

The major product in the biotransformation of lupeol by *M. rouxii* was compound **4** and its relative ratio with respect to lupeol was 26.5% after 10 days incubation. The relative ratio of compound **5** with respect to lupeol was 16.0% on the 7th day remaining unchanged until the 10th day.

Among the filamentous fungi screened for their ability to catalyze the biotransformation of lupeol, *A. ochraceus* and *M. rouxii* were found to be the best catalysts. Although the structural elucidation of the biotransformation products must be further confirmed by NMR studies, our data demonstrated that *A. ochraceus* is a good biocatalyst to introduce double bonds in the lupeol structure, whereas *M. rouxii* exhibits the ability to biocatalyze oxygen insertions into that pentacyclic triterpene. The obtained results open perspectives of using *A. ochraceus* and *M. rouxii* in biotransformations of other pentacyclic triterpenes.

## 3. Experimental

### 3.1. Substrate

Lupeol was isolated from the root bark of *Acosmium dasycarpum* [39]. The physical and spectral data were in agreement with the reported data for this compound [40].

### 3.2. Microorganisms and Maintenance

The following strains were evaluated regarding their ability to biotransform the pentacyclic triterpene: *Aspergillus ochraceus, Chaetomium thermophilum, Humicola grisea* var. *thermoidea*, *Mucor rouxii, Phanerochaete chrysosporium, Scytalidium termophylum* and *Glomerella cingulata*. All the strains belong to a collection of fungi cultures of the Biology Department of the School of Philosophy and Sciences of Ribeirão Preto of the University of São Paulo, except the strain of *Glomerella cingulata*,which belongs to the collection of endophyte fungi of the School of Pharmacy of Ribeirão Preto of the University of São Paulo. The microorganisms are stored as a conidial suspension on silica gel (6–12 mesh, grade 40, desiccant activated) at 4 °C and on slants of solid oatmeal baby food consisting of 0.4% (w/v) oatmeal and 1.8% (w/v) agar.

### 3.3. Biotransformation Procedure

A two-step culture was performed for biotransformation reactions. An initial inoculum of a suspension of 4 × 10^6^ conidia/mL was added into 125 mL Erlemeyer flasks containing 15 mL of seed medium [41]. The flasks were incubated for 48 h at 30 °C on a rotary shaker operating at 120 rpm (preculture), except the flasks containing the cultures of *Chaetomium thermophilum, Humicola grisea* var. *thermoidea* and *Scytalidium termophylum*, which were incubated for 72 h at 40 °C. The resulting mycelia were harvested, rinsed and transferred into 250 mL Erlenmeyer flasks containing 30 mL of Czapek (sucrose 3.0%, NaNO_3_ 0.2%, K_2_HPO_4_ 0.05%, MgSO_4_.7H_2_O 0.05%, KCl 0.05% and FeSO_4_. 7H_2_O 0.001%) or Koch’s K1 (glucose 0.18%, bacteriological peptone 0.06% and yeast extract 0.04%) media. Lupeol was added to each flask as a solution in dimethylsulfoxide (3 mg dissolved in 600 μL). Control flasks consisted of culture medium with fungi without lupeol, culture medium with dimethylsulfoxide without lupeol and fungi, culture medium with fungi and dimethylsulfoxide without lupeol, culture medium with lupeol without fungi, and only culture medium without anything. Biotransformation experiments were carried out at 30 or 40 °C, depending on the fungus, for 240 h with shaking at 120 rpm. Each flask was taken every 24 h, extracted with ethyl acetate, and analyzed by GC-MS. The ratio between the substrate and its transformed products was determined on the basis of the peak area of GC. 

### 3.4. GC-MS Analyses

GC-MS analyses were performed on a Shimadzu GCMS QP2010 Plus instrument equipped with a quadrupole mass analyzer. The following conditions were used: the carrier gas was He at a constant flow of 1.46 mL/min, the column was DB-5MS (30 m × 0.25 mm i.d.; film thickness 0.25 μm, 5% crosslinked phenyl-methylpolysiloxane). The injector temperature was set at 240 °C, with a split ratio of 1:60. The column temperature was programmed from 50 to 280 °C at 10 °C/min; time run, 60 min. The column outlet was inserted directly into the electron ionization source block, operating at 35 eV. The scanned mass range was 50-600 amu. The MS spectra obtained were compared with those of Wiley 7.0 spectra library. 

## 4. Conclusions

Mass spectrometry was demonstrated to be an efficient analytical method to select promising biocatalysts for the compound investigated in this study. The best biocatalysts were found to be *A. ochraceus* and *M. rouxii*. The biotransformation processes were influenced by the culture medium and incubation period.
